# Improvement of skin lesions in corticosteroid withdrawal-associated severe eczema by multicomponent traditional Chinese medicine therapy

**DOI:** 10.1186/s13223-021-00555-0

**Published:** 2021-07-09

**Authors:** Serife Uzun, Zixi Wang, Tory A. McKnight, Paul Ehrlich, Erin Thanik, Anna Nowak-Wegrzyn, Nan Yang, Xiu-Min Li

**Affiliations:** 1 New York Institute of Technology College of Osteopathic, Old Westbury, NY 11545 USA; 2 Department of Allergy, Peking Union Medical College Hospital, Beijing, 100730 China; 3 Department of Microbiology and Immunology, Department of Otolaryngology, School of Medicine, New York Medicine College, 40 Sunshine Cottage Rd, Valhalla, NY 10595 USA; 4 Department of Pediatrics, New York University Langone Health, New York, NY 10029 USA; 5 Department of Environmental Medicine and Public Health, Icahn School of Medicine At Mount Sinai, New York, NY 10029 USA; 6 General Nutraceutical Technology, LLC, 525 Executive Boulevard, Elmsford, NY 10523 USA

**Keywords:** Severe eczema, Corticosteroid withdrawal syndrome, Traditional Chinese Medicine, IgE, Eosinophil

## Abstract

**Rationale:**

We recently showed that multicomponent traditional Chinese medicine (TCM) therapy had steroid-sparing effects in moderate-to-severe eczema. We sought to evaluate TCM effects in severe eczema in a 7-year-old male with refractory disease and corticosteroid withdrawal syndrome.

**Methods:**

Prior to referral, the patient had been treated since infancy with increasingly intensive standard of care, including high-dose topical and systemic corticosteroid and antibiotic therapy and was unable to tolerate further steroid treatment. The patient was administered a combination of oral and topical TCM for 17 months following discontinuation of his steroid regimen. His overall medical condition was assessed by SCORAD criteria and laboratory evaluations of serum IgE, absolute eosinophil count, and liver and kidney function tests.

**Results:**

The patient showed rapid improvement of clinical measures of disease after starting TCM therapy, with marked improvement of sleep quality within the first week, complete resolution of itching, oozing, and erythema at 2 weeks, and a 79% and 99% decrease in his SCORAD values after one month and 3–6 months of TCM, respectively. Serum total IgE decreased by 75% (from 19,000 to 4630 (kIU/L), and absolute eosinophil counts decreased by 60% (from 1000 to 427 cells/μL) after 12 months of treatment. The patient did not require oral or topical steroids during the 17-month trial of TCM. TCM was tapered without complications. His dermatologic manifestations continued to be well-controlled 3 months after discontinuation.

**Conclusion:**

This case study suggests TCM should be further evaluated in controlled clinical studies of patients with severe, refractory eczema and steroid withdrawal syndrome.

**Supplementary Information:**

The online version contains supplementary material available at 10.1186/s13223-021-00555-0.

To the Editor,

Eczema has a worldwide prevalence of up to 20% in children. Topical corticosteroids remain first-line for treatment of eczema; however, 20–30% of patients with chronic eczema are refractory to topical steroids and about 10% of patients with eczema require systemic treatment achieve disease control [1]. Chronic eczema is associated with impaired life quality relating to the physical and emotional suffering caused by sleep disturbance, as well as extreme itching, and painful skin. Development of topical corticosteroid withdrawal syndrome (colloquially known as “steroid addiction”) among patients who chronically use topical corticosteroids is associated with additional difficulty in managing symptoms including burning papulopustules and erythroderma [2]. Steroid-sparing biologics that target Th2-associated cytokines may be effective for a percentage of patients, but they remain costly and are unavailable for use in young children. This situation represents an especially important, unmet healthcare need for steroid-sparing, alternative treatments for eczema.

Treatment for eczema is well documented in Traditional Chinese Medicine (TCM) literature, including acupuncture and internal and external herbal medicine approaches [3]. Recent retrospective studies by our group showed that multicomponent TCM therapy had steroid-sparing effects in both young children and adults with moderate to severe eczema [4]. However, no previous publications have reported whether TCM therapy is effective in improving eczema associated with steroid-withdrawal syndrome and chronic corticosteroid addiction. Here, we present the case of a highly allergic pediatric patient with steroid-refractory chronic eczema and steroid withdrawal syndrome who was successfully treated with multicomponent TCM therapy. Written informed consent to publish this case report and use photos was obtained prior to treatment with TCM therapies.

A 7-year-old male with AD since 2 months of life presents with persistent refractory eczema despite chronic mid-potency topical corticosteroid use and an 18-month trial of step-up therapy to high-potency topical corticosteroids. Over a 6-month period prior to TCM, he had used eight courses prednisolone (15 mg/5 mL oral solution) treatment to control his eczema (10–14 days/course). However, the eczematous lesions returned as soon as 3 days after completing the last course of prednisolone and progressed to total body erythroderma over 12 days, with increased severity compared to that prior to prednisolone use. He was diagnosed with steroid withdrawal syndrome with the complication of *Staphylococcus aureus* infection confirmed by skin culture. He responded poorly to antibiotics (oral cephalexin and topical mupirocin) combined with steroid therapy by injection. His primary allergist referred him to the senior author, who referred him to a secondary allergist and a dermatologist for evaluation before starting TCM therapy at the Integrative Health and Acupuncture clinic in New York.

Upon the first visit for TCM therapy in June 2016, the patient presented with total body erythroderma characterized further by severe erythema, edema, oozing, and excoriations as well as extensive blistering and bleeding (Fig. 1a). Disease was deemed severe based on a score of 103.6 by standardized SCORAD. He was unable to walk secondary to pain and had daytime somnolence related to restless sleep during the night. His medical history was significant for multiple food allergies and allergic rhinitis associated with perennial and seasonal allergen sensitization. He reported uncontrollable pruritus despite taking cetirizine 5 mg/5 mL once daily, oral hydroxyzine HCL (Atarax) 10 mg/5 mL every six hours, and diphenhydramine 12.5 mg as needed. Laboratory results provided by his primary allergist indicated elevated total serum IgE (19,000 kIU/L, normal 100 kIU/L), eosinophilia (1 × 10^3^ cells/µL, normal range 0–0.3 × 10^3^ cells/µL) (Table 1), and high specific IgE levels to multiple food and environmental allergens (not shown). He had positive allergen skin tests to peanut, egg, and cat in 2015. He had a history of anaphylaxis from peanuts, eggs, milk, and seeds, despite a negative milk skin test. Egg causes wheezing, while milk, peanut, soy and wheat caused rash. His aspartate aminotransferase, alanine transaminase and blood urea nitrogen levels were all within normal range.

**Fig. 1:**
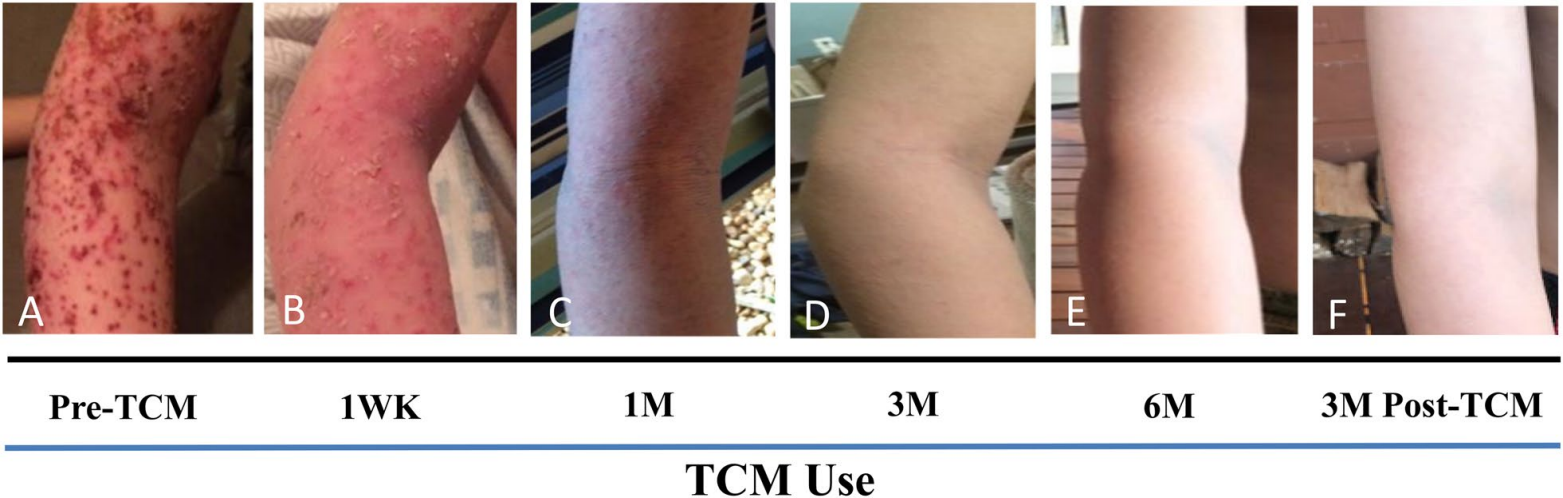
Progress during treatment. **A** 12 days after the last course of prednisone, with MSSA Staph aureus infection and poor response to steroid injection. **B** Improvement of skin lesions by 1 Week of TCM use including internal tea, external herbal bath and creams. **C** 1 month of TCM use, **D** 3 months of TCM use, **E** By 6 months, skin remained to be well controlled. No steroids or antibiotics were used during the course of TCM therapy. **F** Skin reveals no apparent recurrence 3 months after TCM discontinuation

**Table 1:** Laboratory, immunology and safety testing. Blood work was ordered by allergist prior to the TCM visit and 12 months after use of TCM

	Pre-TCM	12M of TCM	Reference range
Total serum IgE (K/µL)	19,000	4630	<100
While blood cells (cells/µL)	11	6.1	4.3–12.4
Hemoglobin (g/dL)	13.7	13.1	10.9–14.8
Platelets (K/µL)	479	382	190–459
Absolute eosinophil (× 10^3^)	1	0.4	0–0.3
Creatinine (mg/dL)	0.54	0.48	0.37–0.62
Aspartate aminotransferase (U/L)	29	26	0–60
Alanine aminotransferase (U/L)	20	12	0–25

The patient received multicomponent TCM therapy of herbal bath additive, herbal creams and internal teas, described previously [4]. Within 1 week of treatment, his lesions showed signs of improvement (Fig. 1b). By 1 month of treatment, his skin lesion intensity was significantly improved; there was no oozing, erythema or excoriation, but with persistence of dryness and lichenification (Fig. 1c). His SCORAD values decreased by 79% (from 103.6 to 21.8) after 1 month of TCM and by 99% following 3–6 months of TCM (Fig. 1d, e; Additional file 1: Figure S1). Most strikingly, he showed rapid improvement in his quality of sleep within 1 week and the patient reported no itching by 2 weeks of treatment. Notably, throughout TCM treatment he did not require use of oral or topical steroids. His total serum IgE decreased 75% (from 19,000 to 4630 kIU/L) by 12 months (Table 1). Absolute eosinophil counts decreased by 60% (from 1 to 0.427 × 10^3^ cells/µL). Liver and kidney function tests remained within normal range (Table 1). His skin remained well-controlled while on maintenance of TCM regimen, and by 17 months of TCM he was able to taper and discontinue the TCM regimen without any flare-up. His skin continued to be well controlled at least 3 months after discontinuation of TCM (Fig. 1f). He even experienced improvement of documented anaphylactic food allergies, including reintroduction of wheat and soy at 6 months post TCM, and successfully passing a milk challenge in 2019, 1 year after completing TCM therapy.

In summary, we present a case of steroid withdrawal syndrome treated with multicomponent TCM in a 7-year-old child with chronic eczema since infancy. In our case, his allergist, dermatologist, and the local Children’s hospital emergency care physicians ruled out hyper IgE syndrome, a genetic disorder with STAT3 mutation, because the patient did not have characteristic facial and dental abnormalities or recurrent lung infections associated with this syndrome. [5, 6] Instead, it is believed that the isolated hyper serum IgE seen on laboratory studies was due to his chronic and severe eczema and overuse of steroids, or steroid withdrawal. As supportive evidence, his IgE was markedly reduced after TCM therapy at the 1-year mark when his skin lesions have markedly improved, whereas syndromic hyper IgE would not resolve with therapy.

We demonstrated multicomponent-TCM therapy markedly improved his skin lesions, sleep disturbance, pruritus, hyper-serum IgE and peripheral blood eosinophil count. The mechanisms might be due to multiple compounds that target on multiple mechanisms related to eczema pathology. Several of the herbs used in this case study have known immunologic effects. The active component of *Radix arnebiae* is shikonin, which has been shown to reduce TGF-β-induced collagen production in scar-derived fibroblasts [7]. *Radix glycyrrhizae* has been shown to inhibit LPS induced NF-κB activation, a key player in AD disease pathology [8], and its compound 7,4 dihydroxyflavone reduces eotaxin-production and Th2 cytokines [9]. *Kochia scopariae*-derived oleanolic acid demonstrated antibiotic properties against *Listeria monocytogenes* [10]. *Flos lonicerae* has antibacterial action against *Staphlococcus aureus*, streptocococci, and *Salmonella typhi*, and exhibits an anti-inflammatory and hepatic cell protective effect [11]. In this case study the mechanisms of action were not studied. However, the multicomponent herbal approach allows for utilization of the anti-inflammatory and anti-microbial properties of these key herbs. In vitro studies have shown that the herbal internal tea used for this patient has anti-IgE, eotaxin and TNF-α effects (data not shown). Previous studies showed it inhibited Th2 cytokine IL-4 production [4]. This report, like any retrospective case report, is limited by recall bias and the fact it represents a single case. However, multicomponent TCM therapy has been reproducible in other patients with steroid withdrawal syndrome. More studies, in particular prospective studies, are needed to investigate the combined effects of the components of TCM for eczema. Large studies are needed to confirm the efficacy of multicomponent TCM for steroid-withdrawal associated severe eczema.

## Supplementary Information

**Additional file 1: Figure S1.** Scoring Atopic Dermatitis (SCORAD) values throughout the course of TCM treatment. (A) Total overall SCORAD values. (B) SCORAD values of regional areas affected by dermatitis. (C) SCORAD values indicating objective findings of dermatitis intensity. (D) SCORAD values indicating subjective findings of pruritus and quality of sleep.

## Data Availability

The data referenced in this study is available in this published article: Improvement of skin lesions and life quality in moderate-to-severe eczema patients by combined TCM therapy by Thanik E, Wisniewski JA, Nowak-Wegrzyn A, Sampson H, Li XM.
