# Supplementary figures and images for: Improvement of skin lesions in corticosteroid withdrawal-associated severe eczema by multicomponent traditional Chinese medicine therapy
Source: Allergy Asthma Clin Immunol. 2021 Jul 9;17:68. doi: 10.1186/s13223-021-00555-0 (PMC8268267; doi:10.1186/s13223-021-00555-0)

## Slide 1
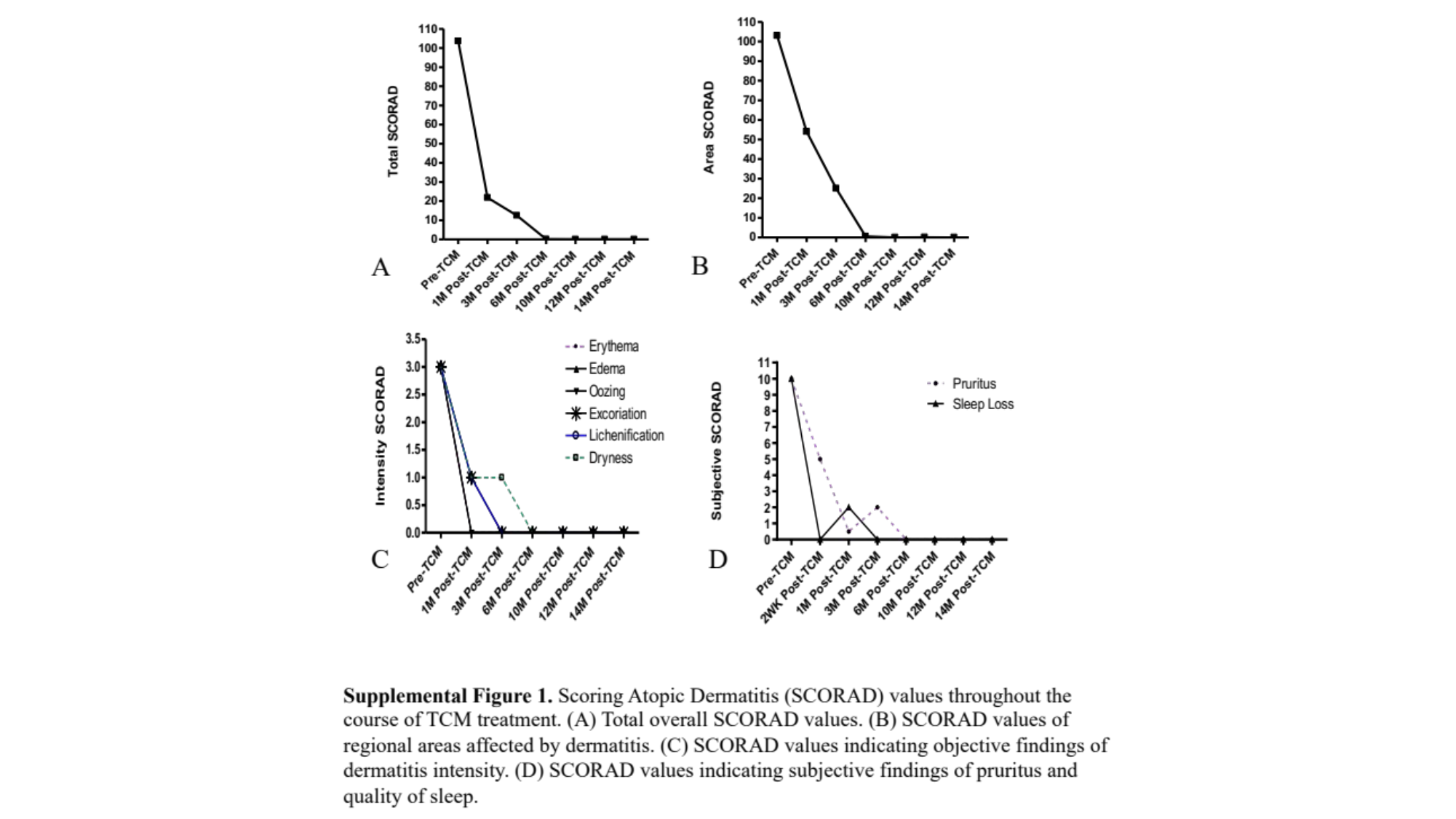

Supplement: Supplementary file 1 — Additional file 1: Figure S1. Scoring Atopic Dermatitis (SCORAD) values throughout the course of TCM treatment. (A) Total overall SCORAD values. (B) SCORAD values of regional areas affected by dermatitis. (C) SCORAD values indicating objective findings of dermatitis intensity. (D) SCORAD values indicating subjective findings of pruritus and quality of sleep. [file 13223_2021_555_MOESM1_ESM.pptx]
